# An Overview of Advancements in Proteomic Approaches to Enhance Livestock Production and Aquaculture

**DOI:** 10.3390/ani15131946

**Published:** 2025-07-02

**Authors:** Jakree Jitjumnong, Anukul Taweechaipaisankul, Jou-Ching Lin, Supatirada Wongchanla, Schwann Chuwatthanakhajorn, Chih-Jen Lin, Luu Tang Phuc Khang, Nguyen Vu Linh, Papungkorn Sangsawad, Nguyen Dinh-Hung, Pin-Chi Tang, Tossapol Moonmanee

**Affiliations:** 1 Department of Animal and Aquatic Sciences, Faculty of Agriculture, Chiang Mai University, Chiang Mai 50200, Thailand; jakree.j@cmu.ac.th (J.J.); khang_luu@cmu.ac.th (L.T.P.K.); linhvu.n@cmu.ac.th (N.V.L.); 2 Functional Feed Innovation Center, Faculty of Agriculture, Chiang Mai University, Chiang Mai 50200, Thailand; 3 Department of Biology, Faculty of Science, Chulalongkorn University, Bangkok 10330, Thailand; anukul.t@chula.ac.th; 4 Department of Animal Science, College of Agriculture and Natural Resources, National Chung Hsing University, Taichung 40227, Taiwan; moveon.cheetah@gmail.com (J.-C.L.); pctang@dragon.nchu.edu.tw (P.-C.T.); 5 Department of Animal Science, University of California, Davis, CA 95616, USA; swongchanla@ucdavis.edu; 6 Faculty of Veterinary Science, Mahidol University, Salaya, Nakhon Pathom 73170, Thailand; schwann@liverpool.ac.uk; 7 Centre for Reproductive health, Institute for Regeneration and Repair (IRR), University of Edinburgh, Edinburgh EH16 4 UU, UK; chih-jen.lin@ed.ac.uk; 8 School of Animal Technology and Innovation, Institute of Agricultural Technology, Suranaree University of Technology, Nakhon Ratchasima 30000, Thailand; papungkorn@sut.ac.th; 9 Aquaculture Pathology Laboratory, School of Animal & Comparative Biomedical Sciences, The University of Arizona, Tucson, AZ 85721, USA; dinhhung@arizona.edu; 10 The iEGG and Animal Biotechnology Center, National Chung Hsing University, Taichung 40227, Taiwan

**Keywords:** aquaculture, biomarkers, livestock production, proteomics, sustainable agriculture

## Abstract

Proteomics, the large-scale study of proteins and their functions, is an essential tool in biological research, particularly in livestock production and aquaculture. This review explores the significance of proteomic techniques and technologies in enhancing agricultural practices. Key methods, including mass spectrometry, two-dimensional gel electrophoresis, and protein microarrays, enable researchers to analyze protein complexity in biological systems. In livestock production, proteomics improves animal health, growth, reproduction, and disease resistance, contributing to more efficient and sustainable practices. In aquaculture, it optimizes fish health, breeding strategies, and feed efficiency, promoting sustainable farming. Despite its potential, proteomics faces challenges, such as complexity, the need for standardized methods, and difficulties in data interpretation. However, emerging advances—including multi-omics integration, real-time monitoring, and improved understanding of protein functions under varying environmental conditions—offer promising solutions. In conclusion, proteomics is poised to transform livestock production and aquaculture, addressing key challenges in food security and sustainable agriculture.

## Simple Summary

Proteomics is a powerful tool transforming both livestock production and aquaculture by providing molecular insights into animal health and productivity. In livestock, it enhances breeding by identifying the biomarkers for reproduction, disease resistance, and productivity. It also improves meat quality, fertility, and feed efficiency, while aiding stress management through biomarkers like heat shock proteins. In aquaculture, proteomics optimizes fish growth, immune responses, and stress adaptation through non-invasive techniques like blood and mucus analysis. It supports disease management by identifying immune markers and disease mechanisms, while also improving reproductive biology and hatchery management through protein markers for egg and sperm quality. Overall, proteomics contributes to more sustainable and efficient practices in both sectors, improving breeding, feed efficiency, welfare, disease control, and environmental adaptation.

## 1. Introduction

Proteomics, which involves the large-scale analysis of proteins within biological systems, emerged in the mid-1990s as a complementary field to genomics [1]. Its primary objective is to comprehensively map the proteome—the complete set of proteins in cells, tissues, or organisms—including their interactions and post-translational modifications [2]. Unlike approaches that focus on individual proteins, proteomics enable high-throughput analysis, providing a holistic understanding of biological systems [2]. The proteome is highly dynamic, influenced by physiological conditions and environmental factors, necessitating advanced analytical methodologies [1]. Standard proteomic workflows typically involve protein separation, mass spectrometry analysis, and bioinformatic-driven data interpretation [3]. The complexity of the proteome is further compounded by factors such as tissue-specific gene expression, alternative splicing, and post-translational modifications, which together result in a proteome that exceeds the number of genes in the genome [4]. Proteomics has become an invaluable tool in biomedical research, particularly for comparative analyses of protein expression between healthy and diseased states, offering critical insights into disease mechanisms [1]. Moreover, its application in pharmaceutical research and novel drug discovery has significantly expanded in recent years [5]. Advances in targeted liquid chromatography–mass spectrometry (LC-MS) have enhanced the accuracy and sensitivity of protein identification and quantification, facilitating progress in various areas of systems biology, such as proteogenomic, interactomics, and kinomics [6]. Despite its promising potential, proteomics faces several challenges, including complexities in sample preparation, protein fractionation, and data interpretation [7]. Mass spectrometry remains the cornerstone of proteomic investigations, with liquid chromatography–tandem mass spectrometry (LC-MS-MS) and matrix-assisted laser desorption/ionization time-of-flight/time-of-flight (MALDI-TOF/TOF) being widely employed [8]. The inherent complexity of proteomes, coupled with the dynamic nature of protein expression and the high costs associated with proteomics infrastructure and specialized personnel, however, limits broader adoption, especially in resource-limited settings [8]. Nevertheless, proteomics continues to drive advances in biomedical sciences by elucidating protein structure, functions, and signaling pathways relevant to various diseases [8].

Beyond human health, proteomics has become an essential tool in livestock and aquaculture research, addressing critical challenges in these rapidly expanding sectors. In aquaculture, proteomics analyses provide a deeper understanding of fish biology, disease diagnosis, stress management, and food security [9,10]. Proteomics facilitate the identification of biomarkers for species differentiation, disease surveillance, and animal welfare assessment [9]. In dairy production, proteomics research supports sustainable practices by improving knowledge of animal health, disease prevention, and management systems [11]. Notably, proteomics plays a crucial role in investigating pathogen–host interactions, making it a promising tool for animal disease research and diagnostics [10].

This review aims to explore the role of proteomics in advancing our understanding of biological systems, with a particular focus on its applications in livestock and aquaculture. This review will discuss proteomics techniques, their specific applications in animal sciences, and the challenges and limitations faced by the field. Additionally, it will highlight the increasing significance of proteomics in enhancing productivity, sustainability, and disease management in these industries while discussing future directions for the continued advancement of proteomic research.

## 2. Applications of Proteomics in Livestock Production

The rapid evolution of molecular biology has significantly transformed livestock breeding, shifting from traditional phenotypic selection to advanced genetic and molecular approaches. Key innovations such as genomic selection and marker-assisted selection (MAS) have revolutionized breeding programs by enhancing genetic improvement and providing deeper insights into economically important traits. High-throughput omics technologies, particularly in genomics and proteomics, have further refined these methods, allowing breeders to make more precise and informed decisions. In animal breeding, genomic selection and MAS have accelerated the identification of genes and quantitative trait loci (QTL) related to production and reproductive traits, facilitating more targeted breeding strategies. Meanwhile, proteomic analysis helps in understanding the molecular mechanisms that govern these traits, offering new avenues for optimizing fertility, meat quality, and overall health. By identifying protein biomarkers associated with reproduction, immunity, and stress response, proteomics has become an indispensable tool for improving livestock management. This chapter explores the impact of genetic improvement and breeding programs, with a particular focus on the role of proteomics in advancing livestock productivity. From enhancing fertility and disease resistance to optimizing meat quality and feed efficiency, molecular tools are shaping the future of sustainable and profitable livestock production.

### 2.1. Genetic Improvement and Breeding Programs

These methods enhance genetic improvement, providing insights into economically relevant traits and underlying biological processes [12]. High-throughput omics technologies have advanced genomic selection, making it a precise and widely accepted approach in animal breeding [13]. Numerous genes and quantitative trait loci (QTL) associated with production and reproductive traits have been identified, facilitating selection and breeding decisions [14]. Proteomics has become integral to understanding the molecular mechanisms underlying key livestock traits, including reproduction, disease resistance, and stress tolerance [15,16,17]. Proteomics supports genetic improvement programs by analyzing protein functions, structures, and interactions [12,18]. Proteomic studies of reproductive processes provide insights into fertility regulation, enabling the early detection of fertility issues and optimization of breeding strategies in vitro [19,20]. Differential protein expression in high- and low-fertility sperm affects capacitation, acrosomal reaction, and sperm–oocyte communication [21]. For instance, the upregulation of metabolic proteins such as pyruvate kinase M2 (PKM2) and ATP5B enhances ATP production essential for sperm motility [18]. Additionally, the increased expression of casein kinase 2 (CKII) and A-kinase anchor protein-4 (AKAP4) in high-fertility sperm improves chromatin decondensation and motility [18].

Proteomic analysis of reproductive fluids provides insights into hormonal regulation, maternal–gamete interactions, and embryonic development [19,22]. Oviductal fluid proteins vary across estrous cycle stages, with heat shock proteins (HSPA5, HSPA8, and HSP90B1) upregulated post-ovulation, influencing embryonic development [23]. NF-κB-related proteins in uterine fluid post-embryo transfer support trophoblast invasion and placental formation [24]. Proteomic analysis of bovine follicular fluid has identified fertility-related proteins, including TIMP2 and complement component C8 alpha chain [25].

**Figure 1:**
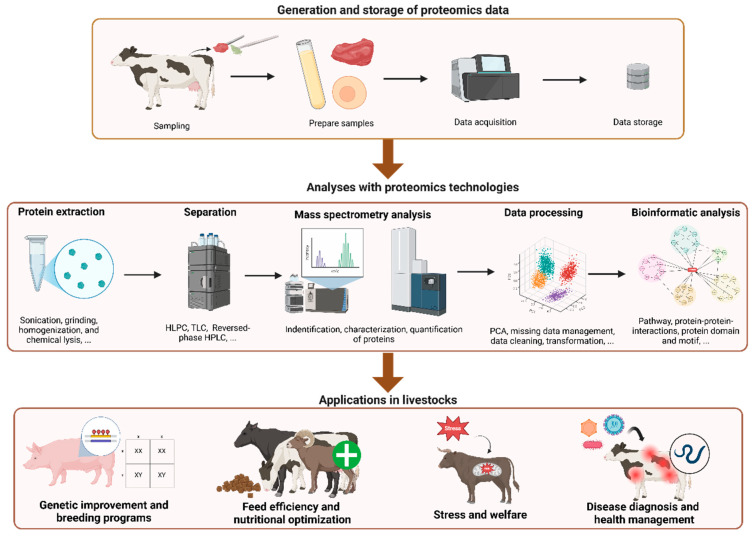
Overview of the proteomics workflow in livestock sciences: from data generation and storage to applications. The upper panel illustrates the process of generating and storing proteomics data, including sampling, sample preparation, data acquisition, and storage. The middle panel highlights the analyses with proteomics technologies, including protein extraction, separation, mass spectrometry, data processing, and bioinformatic analysis. The lower panel demonstrates various applications in livestock, such as genetic improvement and breeding programs, feed efficiency and nutritional optimization, stress and welfare management, and disease diagnosis and health management.

Proteomics also improves meat quality by identifying the biomarkers linked to flavor, tenderness, and juiciness [26] (Figure 1). Protein biomarkers relate to pathways such as energy metabolism, oxidative stress, and structural integrity [27,28,29]. Enzymes like MDH1 and ENO3 are more abundant in tender meat, while ALDH1A1 is associated with toughness [29,30]. Heat shock proteins (HSP1A1, CRYAB, HSP20, and HSP27) are prevalent in tougher meat, whereas MYL1 and MYH1 are linked to tenderness. Additionally, TRIM72 is abundant in tender meat, while FHL1 is higher in tough samples [27].

In dairy science, proteomic analysis identifies biomarkers influencing milk quality, fat content, and lactation efficiency [31,32]. Proteomic variations between colostrum and mature milk reveal immunoglobulin dominance in colostrum, while antigen-specific proteins such as CLU and MFGE8 are prevalent in mature milk [33]. Furthermore, proteomics assesses protein modifications in dairy processing, including lactosylation changes in casein and β-lactoglobulin due to ultra-heat treatment and pasteurization [34].

### 2.2. Feed Efficiency and Nutritional Optimization

Feed accounts for approximately half of livestock production costs, making feed efficiency a key factor in reducing expenses and improving profit margins [35]. Residual feed intake (RFI) is a widely used measure that identifies animals consuming less dry matter than expected relative to their body weight and growth, thereby reducing feed costs [36,37]. However, RFI studies are complex due to their dependence on intricate biological mechanisms regulated by multiple genes affecting physiological and metabolic functions [37]. Instead, omics technologies provide insights into feed efficiency by analyzing metabolic and proteomic changes [38,39].

Feed efficiency is influenced by dietary composition, management, and metabolic states [40]. Studying RFI often requires invasive techniques, such as liver biopsy, due to the liver’s central role in metabolism [41,42]. Metabolomic and proteomic liver analyses reveal that high-forage diets in dairy heifers significantly alter protein abundance, affecting fatty acid oxidation, ketone synthesis, amino acid metabolism, gluconeogenesis, and oxidative stress defense [39]. Lipid metabolism is crucial for energy balance, as lipids provide an alternative energy source during metabolic stress [43]. High-forage diets upregulate the mitochondrial enzymes (ACSS3, ECHS1) and peroxisomal enzymes (ACOX1) involved in fatty acid breakdown [39,44]. Increased fatty acid oxidation also enhances ketone body synthesis, providing alternative energy [45]. Additionally, oxidative defense proteins (CYP450s, SOD1, GSTMs, TXN) increase in high-forage-fed heifers, likely due to reactive oxygen species from metabolic processes, contributing to reduced energy efficiency [46,47].

Beyond invasive sampling, few studies have explored the semi-invasive and non-invasive methods for proteomic analysis of RFI traits. Notably, high-RFI cattle showed increased TGF-β levels, a key regulator of feed efficiency [48]. Additionally, low-RFI cattle exhibited higher levels of metabolic hormones (KNG1, FBN1, ADIPOQ), which influence feed intake, energy metabolism, and lipid accumulation [49,50]. Non-invasive saliva proteomics offers insights into both host and microbial proteins, improving understanding of animal–microbiota interactions [51]. Salivary proteomics may also provide reliable health biomarkers, serving as an alternative to blood-based analysis [52,53,54].

### 2.3. Stress and Welfare

The study of stress and welfare in livestock is of paramount importance due to its direct impact on animal health, productivity, and ethical considerations in animal husbandry. Proteomics, as a cutting-edge approach, plays a pivotal role in this field by enabling the identification of protein biomarkers that reflect the physiological responses of livestock to stress and welfare conditions (Table 1). Stress in livestock, whether physiological or environmental, can lead to adverse effects on immune function, growth, reproduction, and overall well-being, ultimately affecting the quality and efficiency of animal production systems. Recent findings have reported the application of proteomics on animal welfare and stress detection in various species of livestock. For instance, a study by Marco-Ramell and colleagues identified cytoplasmic β-actin as a key protein associated with stocking density stress in pigs. Elevated serum actin levels are suggested to serve as a marker of cell or tissue damage associated with overcrowding conditions [55]. Likewise, a multi-omics analysis of chicken thigh muscle has revealed elevated corticosterone levels and reduced hexose phosphate abundance in response to road transport-induced stress, concluding that road transport impacts cytoskeletal structure and carbohydrate metabolism [56]. Heat stress is a major challenge to livestock welfare, particularly in regions with extreme temperatures. In pigs experiencing acute heat stress, proteins associated with glycolysis, glycogenesis, and glycogenolysis were markedly upregulated. In addition, elevated levels of manganese superoxide dismutase (SOD) and reduced abundance of peroxiredoxin 2 were identified as significant biomarkers for stress assessment [57]. A study investigating the effects of heat stress on the liver proteome of Peking ducks (*Anas platyrhynchos*) and Muscovy ducks (*Cairina moschata*) revealed the overexpression of heat shock proteins 70 and 10 in both species as a response to heat stress. Moreover, proteins such as α-enolase and S-adenosylmethionine synthetase exhibited species-specific expression patterns, underscoring distinct physiological responses to heat stress [58]. Heat stress in dairy cows has been linked to alterations in serum protein profiles, including increased levels of heat shock protein 70 (HSP70) and reduced concentrations of metabolic proteins such as albumin. These changes serve as biomarkers for heat stress, helping farmers implement management strategies like cooling systems and dietary adjustments [59,60,61]. Monitoring nutritional stress is crucial for maintaining animals in optimal conditions that support their health and prevent unnecessary suffering. Negative energy balance (NEB) is a common metabolic challenge in early lactating cows, arising from insufficient nutrient and energy intake to support the high demands of milk production. This imbalance triggers excessive lipolysis, resulting in the release of free fatty acids into the circulatory system, ultimately contributing to metabolic disorders such as ketosis and fatty liver [62]. Plasma proteomic profile have indicated that increases in acute phase proteins (APPs) such as haptoglobin, a-1-acid glycoprotein (AGP), and a-chymotrypsin and a decrease in Apolipoprotein-AIV and a-2-HS-glycoprotein were highly related to ketosis in cows [63,64]. Furthermore, stomatin and galactose-1-phosphate were reported as key indicators of NEB in milk [65,66]. These insights allow for the development of evidence-based management strategies aimed at improving animal welfare, enhancing disease resilience, and fostering sustainable livestock production.

**Table 1:** Proteomics applications for stress and welfare in livestock.

Indicators	Proteomics Techniques/Method	Species	Findings	References
Stress response	LC-MS/MS	Swine	Label-free quantitative proteomics identified 66 differentially abundant proteins between non-stressed and stressed pigs, with 30 increased and 36 decreased in the non-stressed group.	[67]
Pre-slaughter stress	OFFGEL, SDS-PAGE and LC-MS	Bovine	Five protein bands showed significant differences between normal and DFD meat samples, containing actin, phosphoglucomutase-1, alpha-crystallin B, heat shock protein beta-6, and heat shock protein beta-1.	[68]
Chronic lameness	LC-MS/MS and SDS-PAGE	Bovine	Chronic inflammatory lameness in dairy cows is associated with increased expression of stress proteins with chaperone, metabolism, redox, and structural functions in the dorsal horn of the spinal cord.	[69]
Heat stress	LC-MS/MS	Swine	Overexpression of HSP70 in intestinal epithelial cells led to changes in the expression of many proteins involved in cell–extracellular matrix interactions, cell adhesion, and apoptosis.	[70]
Environmental enrichment	iTRAQ and LC-MS/MS	Swine	Pigs in enriched environments had lower plasma cortisol and lactate levels, indicating a reduced stress response. Pigs in enriched environments showed changes in neurotransmitter levels in the brain, including decreased noradrenaline and dopamine, and increased serotonin, also suggesting a lower stress response.	[71]
Slaughter methods	MALDI-TOF MS	Poultry	Ritual slaughter without stunning resulted in significantly elevated stress indicators like cortisol and triiodothyronine compared to commercial slaughter with electrical stunning.	[72]
Electrical stunning	2-DE and MALDI-TOF/TOF MS	Sheep	The study found 243 proteins that were significantly differentially expressed between stunned and non-stunned (halal) slaughter, with 119 being upregulated and 124 being downregulated.	[73]
Chronic circadian disruption	LC-MS/MS	Bovine	Dairy cows exposed to circadian rhythm disruption during late gestation showed increased markers of oxidative stress and metabolism in their muscle tissue.	[74]
Response of bovine granulosa cells to acute heat stress	LC-MS/MS	Bovine	Heat stress triggered oxidative stress-mediated apoptosis in bovine granulosa cells (bGCs), but the cells exhibited a time-dependent recovery of proliferation potential by 48 h. The study identified 37 differentially regulated metabolites in bGCs in response to acute heat stress, which were involved in bioenergetics support mechanisms and cellular adaptations.	[75]

### 2.4. Disease Diagnosis and Health Management

Proteomics has emerged as an indispensable tool in the diagnosis and management of diseases in livestock production (Table 2). This field focuses on the large-scale study of proteins, which are crucial for understanding the biological processes and disease mechanisms. The application of proteomics in veterinary medicine is particularly relevant for identifying biomarkers that can facilitate early disease detection, monitor health status, and improve overall livestock management practices. This approach offers immense potential for disease detection by enabling the identification of biomarkers critical for diagnosing and monitoring diseases in domestic animals, by utilizing panels of disease-related proteins rather than relying solely on single-protein assessments [76,77]. The analysis of protein profiles from tissues or bodily fluids allows for the precise identification of disease-specific protein signatures. Moreover, this method serves as a cornerstone for livestock health monitoring, providing a comprehensive understanding of protein expression patterns linked to both normal physiological states and pathological conditions. In cattle, acute-phase proteins such as serpin A3-1, vitronectin-like protein, and complement factor H have been identified as early indicators of subclinical mastitis. Similarly, haptoglobin and apolipoprotein A-I have been recognized as key biomarkers of clinical mastitis, a critical disease affecting animal health and milk production [78,79]. In addition, the promising application of proteomics for mastitis detection was highlighted, demonstrating differential protein expression between the E. coli and S. aureus infection groups [80]. Furthermore, variations in serum Interleukin 8 (IL-8), Interferon-gamma (IFN-γ), transforming growth factor β1 (TGF-β1), and overall haptoglobin concentration patterns have been identified among dairy cows infected with different S. aureus strains, enhancing the capacity to identify pathogenic strains at the strain level in mastitis cases [81]. Proteomic approaches for disease detection have also been successfully applied in swine. Elevated levels of alpha-1-acid glycoprotein and complement factors have been identified as key biomarkers for the early detection of porcine reproductive and respiratory syndrome virus (PRRSV) [82]. Comparatively, it has been reported that the appearance of apolipoprotein A-IV precursor, haptoglobin, and the probable chemoreceptor glutamine deamidase cheD were found after foot and mouth disease virus (FMDV) infection in piglets [83]. Additionally, studies have underscored protein expression patterns associated with diseases in pigs including classical swine fever and peritonitis-induced sepsis using proteomic approaches [84,85]. In poultry, proteomic profiling has identified elevated levels of heat-shock proteins (HSP70 and HSP90) in blood plasma as early indicators of Salmonella infection in chickens [86]. Beyond disease detection, proteomic profiling is also applied in gut health monitoring [87]. A study on broiler chickens found that serum citrulline, IFN-ɤ, and cloacal IgA are key candidate biomarkers for identifying intestinal inflammation, which significantly affects feed efficiency, growth, and intestinal barrier function [88]. Likewise, several proteins associated with inflammation, serum leakage, and tissue damage have been identified as critical markers for intestinal barrier damage [89]. These findings highlight the utility of proteomics in early disease detection, offering livestock producers the ability to implement targeted treatments and biosecurity measures.

Proteomics also contributes to the assessment of feed efficiency by identifying biomarkers linked to metabolic and proteomic changes, thereby reducing feed costs. In slow-growing Korat chickens, the label-free quantitative proteomic analysis of duodenal tissue revealed differentially abundant proteins associated with feed efficiency. The study identified 40 proteins involved in pathways such as glycolysis/gluconeogenesis and oxidative phosphorylation, suggesting their potential as biomarkers for selecting chickens with higher feed utilization efficiency [90]. Similarly, research on Nellore cattle using liver proteomics identified 42 proteins with differential abundance between high and low feed efficiency groups, highlighting pathways like glycolysis/glucogenesis and lipid metabolism. These findings demonstrate the potential of proteomics in developing biomarkers for early identification of efficient animals, ultimately optimizing beef production [91]. Collectively, these studies show that proteomic and metabolomic analyses are powerful tools for identifying biomarkers associated with feed efficiency, contributing to reduced feed costs and improved sustainability in livestock production.

**Table 2:** Proteomics applications for disease diagnosis and health management in livestock.

Indicators	Proteomics Techniques/Method	Species	Findings	References
Neosporosis	LC-MS/MS	Bovine	*Neospora caninum* infection primarily impacts the host cell’s mitochondrial processes and metabolism. The low-virulence isolate Nc-Spain1H had a greater influence on the host cell proteome compared to the high-virulence isolate Nc-Spain7.	[92]
*Salmonella* infection	LC-MS/MS	Poultry	*Salmonella* infection increased the abundance of proteins involved in the host’s response to oxidative stress, amino acid metabolism, and lysosomal activity in the spleen of broiler chickens. *Salmonella* infection decreased the abundance of proteins involved in cell cycle progression, RNA binding, and cytoskeletal development in the spleen of broiler chickens.	[93]
Pneumonia and mastitis	2-DE and MALDI-TOF mass spectrometry	Bovine	Identified 60 secreted proteins from *Mycoplasma bovis*, a pathogen that causes pneumonia and mastitis in cattle, and 8 of these proteins were predicted to be virulence-related factors.	[94]
*Mycobacterium avium paratuberculosis* infection	label-free LC-MS/MS	Bovine	Cows resistant to MAP infection showed higher abundance of TLR2 and MHC class II proteins in their PBMCs, indicating a successful defensive immune response. Cows persistently infected with MAP showed higher abundance of ITGA2B and KCNMA1 in their PBMCs, suggesting an unsuccessful immune response.	[95]
Ketosis	LC-MS	Bovine	The metabolomic analysis showed that cows with clinical ketosis had significant alterations in pathways related to amino acid, carbohydrate, and nucleotide metabolism compared to healthy controls, and these changes were consistent across the transition from prepartum to postpartum.	[96]
*Toxoplasma gondii* infection	iTRAQ labeling, and LC-MS/MS	Pig	Overexpression of two potential anti-*T. gondii* proteins, HSP70.2 and PDIA3, in swine macrophage cells enhanced resistance to *T. gondii* infection.	[97]
Mastitis	MALDI-TOF mass spectrometry	Goat	Identified the Staphylococcus species present in 19 isolates from subclinical caprine mastitis, with S. epidermidis being the most common at 47.36%. Henotypic resistance testing showed high resistance to penicillin G (58%), but lower resistance to cefoxitin and oxacillin (both 26.31%). All strains were susceptible to amoxicillin + clavulanic acid.	[98]
Coinfection with Marek’s disease virus and reticuloendotheliosis virus	Tandem mass tag (TMT) labeling and LC-MS/MS	Chicken	MDV and REV coinfection increased viral replication compared to single infections. Coinfection led to differential expression of 98 proteins, with 38 upregulated and 60 downregulated.	[99]
Rotavirus infection	iTRAQ and LC-MS/MS	Pig	Identified 223 differentially accumulated proteins (DAPs) in porcine rotavirus (PoRV)-infected IPEC-J2 cells compared to mock-infected cells, with 125 being up-accumulated and 98 being down-accumulated.	[100]
Deltacoronavirus	iTRAQ and LC-MS/MS	Pig	A total of 78 differentially expressed proteins (DEPs) were identified in IPEC-J2 cells infected with porcine deltacoronavirus (PDCoV), with 23 being upregulated and 55 being downregulated.	[101]
Mastitis	MALDI-TOF MS	Bovine	MALDI-TOF MS fingerprinting was superior to 16S rRNA gene sequencing for discriminating between different streptococcal species and subspecies involved in bovine mastitis. MALDI-TOF MS analysis identified three specific protein biomarkers characteristic of the Streptococcus genus and showed variability at both the species and subspecies level.	[102]

## 3. Applications of Proteomics in Aquaculture

Proteomics, the large-scale study of proteins, has become an indispensable tool in advancing our understanding of fish physiology, particularly in the context of aquaculture. This technology allows researchers to identify key biomarkers associated with various critical traits in aquatic animals, including growth performance, muscle development, immune response, and stress adaptation. By providing insights into the molecular mechanisms underlying these traits, proteomics supports key areas of aquaculture, such as nutrition optimization, farm management, disease control, and food security. The ability to analyze proteins from non-invasive samples, such as mucus and blood, enables researchers to study immune responses, metabolic functions, and reproductive processes without sacrificing fish, making it an ethical and sustainable approach for monitoring fish health and development. Furthermore, proteomic techniques like LC-MS/MS, 2D-PAGE, and MALDI-TOF/TOF offer high-resolution mapping of protein dynamics across different tissues and conditions, enhancing our ability to understand how environmental factors, diets, and diseases affect fish. As aquaculture continues to evolve, proteomics plays a vital role in improving both the quality of farmed fish and the sustainability of aquaculture practices by providing molecular insights that drive improvements in fish health, welfare, and production efficiency.

### 3.1. Proteomic Insights into Fish Physiology

**Figure 2:**
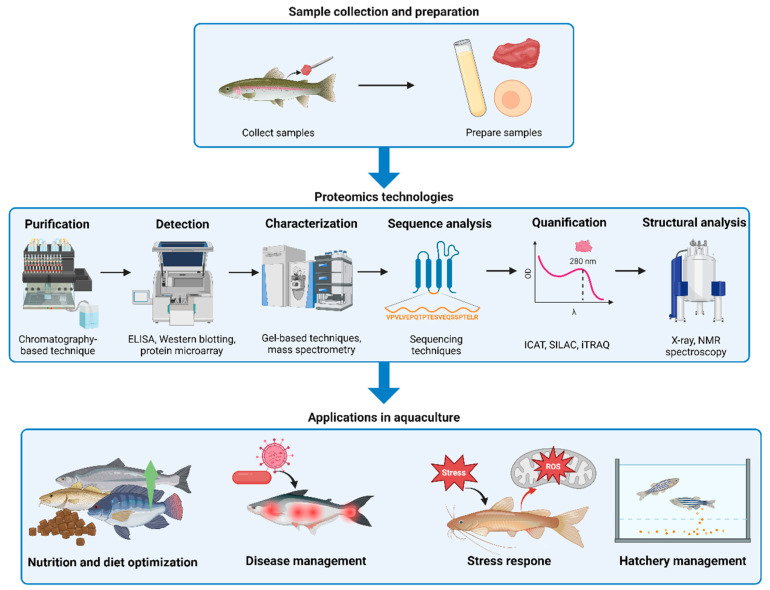
Proteomics workflow in aquaculture: from sample collection to applications. The upper panel outlines sample collection and preparation, involving the collection of aquaculture species samples and subsequent preparation steps. The middle panel describes proteomics technologies, including purification, detection, characterization, sequence analysis, quantification, and structural analysis using techniques like chromatography, ELISA, mass spectrometry, and spectroscopy. The lower panel illustrates the broad applications in aquaculture, such as nutrition and diet optimization, disease management, stress response analysis, and hatchery management.

Proteomics has been employed to identify key biomarkers associated with growth performance, muscle development, immune response, and other critical traits that enhance health and production in aquatic animals [9]. It plays a significant role in various aspects of aquaculture, including nutrition and diet optimization, quality control, farm management, and food security [103,104,105] (Figure 2). Proteomics can be applied non-invasively, using samples such as mucus and blood, to identify proteins involved in immune responses, reproduction, stress adaptation, and metabolic functions without sacrificing fish (Table 3). For example, mucus proteomics stimulate innate immune responses and stress indicators such as galectin-1 and heat shock proteins [106], whereas body fluids (e.g., blood and seminal plasma) reveal proteins linked to reproduction and oxidative stress [107]. Meanwhile, analyses of internal organs, including muscles and lymphoid tissues, provide insights into biochemical responses to environmental and dietary variations [108,109].

**Table 3:** Proteomics applications in monitoring fish health.

Indicators	Technique Used	Species	Findings	References
Immune and stress biomarkers in skin mucus	2D-PAGE, LC-MS/MS	*Gadus morhua*, *Cyclopterus* *lumpus*	Identified immune-related proteins such as galectin-1, cystatin B, heat shock proteins, and peroxiredoxin1.	[106,110]
Innate immunity and physiological status	LC-MS/MS, 2-DE	*Sparus aurata*	Key proteins (e.g., actins, glycolytic enzymes, heat shock proteins) linked to immune defense, metabolism, and stress responses.	[111,112]
Antimicrobial and proteolytic activity	Nano-LC-MS/MS	*Scorpaena plumieri*	Identified 391 proteins with antimicrobial and venom-related activity, including proteolytic enzymes.	[113]
Feeding mechanisms of bloodsucking fish	LC-MS/MS, 1D SDS-PAGE	*Lampetra morii*	Novel proteins involved in blood coagulation suppression and host immune evasion during feeding were identified.	[114]
Reproductive processes in seminal plasma	HPLC-ESI-MS/MS, 2D-DIGE	*Oncorhynchus mykiss*, *Cyprinus carpio*	Identified proteins regulating sperm motility, membrane stability, antioxidative defense, and inflammation responses.	[107,115]
Growth and muscle development	DIGE, MALDI-TOF/TOF	*Sparus aurata*	Proteins such as parvalbumins and Wap65 were linked to growth, stress adaptation, and dietary influences.	[116,117]
Gill proteome analysis	Data-independent acquisition	*Gasterosteus aculeatus*	Explored molecular differences in gill proteins among ecotypes, revealing adaptation to environmental and morphological variations.	[118]
Lymphoid organ function	Shotgun proteomics	*Oncorhynchus mykiss*	Profiling of head kidney and spleen proteins provided insights into immune mechanisms and DNA repair processes.	[119]
Confirmation of specific proteins	LC-MS/MS	*Gadus morhua*	Verified the presence of natterin-like proteins in skin, liver, and kidney, linked to immune responses.	[120]

### 3.2. Disease Resistance and Immunity

Diseases significantly influence fish health, a critical component of aquaculture welfare, and can impact fish production as well as human health through the food chain [121]. Maintaining fish health is essential for ensuring the quality and safety of aquaculture products. Proteomics plays a pivotal role in understanding the disease pathogenesis and immune responses in fish by identifying protein markers involved in immune regulation [10]. For instance, a proteomic analysis of skin mucus from common carp (*Cyprinus carpio*) infected with *Ichthyophthirius multifiliis* revealed the overexpression of metabolic proteins and the downregulation of cytoskeletal proteins, indicating impaired wound healing during the parasitic infection [122]. In *Streptococcus agalactiae*-infected tilapia, proteomic and transcriptomic studies highlighted the pathogen’s activation of toll-like receptor pathways to evade the immune system, while the host relied on NOD receptor signaling to combat the infection [123]. Similarly, the proteomic profiling of rainbow trout (*Oncorhynchus mykiss*) infected with Aeromonas salmonicida demonstrated the upregulation of acute-phase response proteins and complement system components [124], while phosphoproteomic analysis identified fibrinogen alpha chain (FGA) proteins as significant in the infection response [125].

Proteomics also aids in understanding skeletal deformities in fish. For example, proteins such as myosin light chain (MYL), apolipoproteins, creatine kinase M-type (CKM), and calmodulin have been associated with skeletal deformities in rainbow trout larvae [126]. Furthermore, immunoproteomics has been instrumental in vaccine development, particularly in identifying immunogenic outer membrane proteins (OMPs). For instance, OMPs such as YaeT, GroEL, and GldJ were identified as potential antigen candidates for protecting fish against *Edwardsiella* and *Flavobacterium* infections [127]. A study of the Chinese vaccine strain (J-1) of *Aeromonas hydrophila* found that Omp38 is a key antigen that enhances both specific and nonspecific immunity in Chinese breams, suggesting its potential as a vaccine candidate [128]. By integrating proteomics and immunoblotting, researchers have identified virulence-related and antigenic proteins in fish and bacterial pathogens. For example, an analysis of outer membrane proteins from *Vibrio harveyi* identified 11 specific and 4 nonspecific immunogenic proteins in orange-spotted grouper (*Epinephelus coioides*) sera [129]. Similarly, studies on *Francisella noatunensis* in Nile tilapia (*Oreochromis niloticus*) provided insights into host–pathogen interactions [130]. These proteomic approaches offer a molecular-level understanding of disease mechanisms, facilitating targeted disease control strategies and advances in aquaculture health management.

### 3.3. Environmental Adaptation and Stress Response

Stresses, similar to that experienced by higher vertebrates, significantly impact fish growth and overall health. Climate change exacerbates stress in fish, with contributing factors including fluctuations in temperature, pH, oxygen and carbon dioxide levels, handling methods, overcrowding, and osmotic conditions [131]. Enhancing welfare standards in aquaculture requires effective stress monitoring and optimization of fish stress responses [132]. Understanding stress responses is critical for aquaculture sustainability, fisheries management, and fish welfare [131]. Effective stress management and improved disease resistance are essential for sustainable aquaculture development. Advances in genetic methodologies, including transcriptomics and genomics, provide insights into stress responses, physiological adaptations, and disease resistance mechanisms [133]. Stress management strategies in aquaculture involve optimizing environmental conditions, adopting appropriate handling practices, and utilizing genetically improved stocks [131]. Proteomics plays a crucial role in assessing stress at the molecular level by analyzing protein expression and metabolic processes, to develop strategies for mitigating or preventing adverse effects (Table 4). Proteomics studies have identified proteins associated with energy metabolism, immune function, and signaling pathways that exhibit differential expression under physical and chemical stress. These findings highlight the interplay between the immune and osmoregulatory systems in facilitating fish adaptation to environmental fluctuations [134,135].

**Table 4:** Application of proteomics in physical and chemical stress agents in aquaculture.

Stressors	Technique Used	Species	Findings	References
**Physical stress**				
Overcrowding	LC-MS/MS, 2-DE	*Sparus aurata*	Liver and immune proteins showed significant changes under overcrowding compared to optimized rearing conditions.	[136,137]
	2-DE	*Salmo salar*	Proteins in muscle and plasma revealed secondary and tertiary stress responses.	[138]
X-ray irradiation	LC-MS	*Salmo salar*	Persistent alterations in gill proteins observed in early-stage rainbow trout.	[139]
Freezing conditions	2-DE, MALDI-TOF/TOF MS	*Cyprinus carpio*	Sperm proteins supported antioxidative defense, membrane stability, and motility control.	[140,141]
High temperature	LTQ-Orbitrap XL	*Salmo salar*	Proteomic changes in the liver indicated reduced energy expenditure to cope with oxidative stress.	[142]
Low temperature	iTRAQ	*Takifugu fasciatus*	Enhanced oxidative stress response and mitochondrial enzyme activity were linked to cold tolerance.	[143]
Hypersalinity	LC-MS	*Unspecified*	Differentially expressed proteins supported osmoregulation, digestion, and mineral regulation in response to salinity changes.	[144]
High CO2 and temperature	2-DE, Nanoflow LC-MS/MS	*Hippoglossus hippoglossus*	Energy metabolism proteins in gills and immune proteins in blood plasma were significantly affected.	[145]
**Chemical stress**				
Copper	2D-DIGE, iTRAQ	*Oncorhynchus mykiss*, *Cyprinus carpio*, *Carassius auratus gibelio*	Oxidative stress markers, such as superoxide dismutase and cytochrome c, were identified in gills.	[146]
Arsenic	2D, MALDI-TOF/TOF MS	*Labeo rohita*	Identified biomarkers (Apo-A1, A2ML, Wap65) indicate arsenic-induced liver toxicity.	[147]
Benzotriazole	2-DE, TOF/TOF MS/MS	*Gobiocypris rarus*	Neurological and liver alterations differed between male and female fish.	[148,149]
PAHs (Polycyclic Aromatics)	LC-MS/MS	*Gadus morhua*	An albumin-like protein in plasma was linked to PAH-induced stress.	[150]
PCB (Polychlorinated Biphenyl)	MALDI-TOF MS, MS/MS	*Gadus morhua*	Proteins linked to neurotoxicity and stress pathways (e.g., Notch signaling) were identified.	[151]
Bisphenol A	LC-MS/MS	*Danio rerio*	Proteomic changes in brain tissue suggested complex toxicity mechanisms involving metabolism and transport.	[152]
Pesticides (Permethrin, Terbufos)	LC-MS/MS	*Pimephales promelas*	Enrichment of proteins associated with proteasome systems and glycolysis was observed.	[153]
Pesticide (Dieldrin)	LC-MS/MS, iTRAQ	*Danio rerio*	Mitochondrial proteins showed links to diseases like Parkinson’s and Huntington’s.	[154]
Herbicide (Ametryn)	SDS-PAGE	*Danio rerio*	Induced proteins were linked to glycolysis and lipid transport, while suppressed proteins were associated with oxygen transport.	[155]

### 3.4. Reproductive Biology and Hatchery Management

Effective breeding and hatchery management are critical for the sustainability of aquaculture production. Key components include broodstock management, reproductive control, and genetic enhancement [156]. Advances in hatchery technology have significantly improved production through innovations such as formulated broodfish diets, GnRH-induced spawning, and enhanced micro-diets for larvae [157]. Hormonal manipulations are often required to induce oocyte maturation and spermiation in captive fish [158]. Breeding programs play a vital role in enhancing productivity and sustainability. Hatcheries are indispensable for providing high-quality juvenile fish, which are essential for sustainable fish production [159]. Proteomics has emerged as a valuable tool for investigating the molecular mechanisms underlying reproduction and development in fish. Research in fish proteomics has primarily focused on gametogenesis and the development of novel biomedical applications, significantly influencing aquaculture and fisheries. High-quality seeds lead to healthier individuals, and understanding the molecular basis of reproduction supports advances in aquaculture. Egg quality is a critical determinant of reproductive fitness in aquatic species [160]. Proteomic studies have identified putative protein markers associated with oocyte quality, such as vitellogenin, ApoA1, and MBL proteins, in the ovarian fluid of rainbow trout, using shotgun proteomics [161]. In captive hapuku (*Polyprion oxygeneios*), iTRAQ-based analyses identified HSP-70 and IDS proteins as potential biomarkers for assessing egg quality [162]. Proteomic studies of sperm and seminal plasma have also gained importance due to their role in sperm quality and fertilization success [163]. Cryopreservation protein markers are essential for the long-term ex situ conservation of germplasm. Proteomic analyses using 1DE and 2DE have investigated alterations in the semen proteome of rainbow trout induced by cryopreservation, aiding in identifying biomarkers for cryoinjury and high-quality germplasm [164]. Fish roe and caviar, derived from fish eggs, are highly nutritious sources of proteins and lipids. Caviar, particularly from sturgeon eggs, is the most expensive, while roe is also harvested from trout, salmon, carp, and other species [165]. Molecular-level studies of fish gametes have provided insights into reproductive biology and help identify quality markers for these food products. The proteome profiling of female sturgeon ova using 2DE and MALDI-TOF-TOF has provided a foundation for identifying molecular markers related to caviar quality [166]. Findings from proteomic studies have contributed to a deeper understanding of reproductive success in fish and help preserve the quality and integrity of these valuable food sources.

## 4. Challenges and Limitations in the Use of Proteomics in Livestock and Aquaculture Production

Although proteomics has become invaluable in livestock and aquaculture research, offering critical insights into growth, reproduction, disease resistance, and environmental adaptation, its application still faces significant challenges and limitations that hinder its full potential in these fields.

### 4.1. Management and Maintenance of Proteomics Data

A major challenge in proteomics research is managing and maintaining vast datasets. Meticulous data processing is essential for preventing errors and biases during analysis. For instance, proteomics has been applied to study milk protein composition in sheep (*Ovis aries*) and dairy cattle (*Bos taurus*), requiring stringent normalization and scaling techniques to account for individual variability in milk yield and quality [167,168,169]. Similarly, in aquaculture species like Nile tilapia (*Oreochromis niloticus*), proteomics has been used to analyze the liver and muscle proteins under various feeding regimes [170,171]. However, the absence of robust, species-specific proteomics databases restricts the scope of such studies. While databases like ASlive have been developed to catalog alternative splicing events in livestock, equivalent databases for aquaculture species remain underdeveloped, limiting the identification of novel proteins and biomarkers [12].

### 4.2. Lack of Comprehensive Phenomics Data

The lack of detailed phenomics data—comprehensive organism-wide phenotypic information—hinders the accurate interpretation of proteomics data. Key traits such as adaptability, fitness, disease resistance, and production performance should be systematically recorded in livestock and aquaculture species [172]. For example, in *Gallus gallus domesticus*, proteomics could be used to study stress biomarkers during heatwaves [173]. However, inconsistent phenomic data, including body temperature and feed intake, limit comprehensive analysis. Similarly, species such as Pacific white shrimp (*Penaeus vannamei*) and Atlantic salmon (*Salmo salar*) could benefit from detailed growth and disease resistance data under varying environmental conditions [174].

### 4.3. Sample Preparation and Variability

Sample preparation introduces significant variability in proteomics studies. Protein extraction, purification, and fractionation vary across protocols, equipment, and environmental conditions, affecting reproducibility. While gel- and MS-based techniques have been successfully applied in many organisms, quantifying aquatic protein hydrolases remains challenging due to their inherent complexities. In zebrafish (*Danio rerio*), proteomics studies of the reproductive system often require multiple validation techniques to ensure reliable results [175,176]. In livestock, biological sample heterogeneity—such as blood plasma from different breeds of cattle or pigs—complicates protein biomarkers [177,178]. Additionally, the clinical stage of a disease influences protein expression patterns, as observed in the urinary proteomics of cattle infected with bovine spongiform encephalopathy (BSE), underscoring the need for careful sample selection and study design [179].

### 4.4. Species-Specific and Tissue-Specific Challenges

Proteomic analyses must address species- and tissue-specific challenges. For instance, aquatic protein hydrolases are often contaminated with nonprotein substances such as polysaccharides, complicating protein extraction and analysis [180]. Similarly, the protein composition of blood plasma in cattle differs significantly from that of muscle tissue, necessitating customized preparation methods for each sample type [177,181].

### 4.5. Cost and Accessibility

The high costs of proteomics research and technical variability in methodologies hinder its widespread adoption, particularly in developing regions where livestock and aquaculture are economically important industries. For example, mass spectrometry for protein analysis remains prohibitively expensive for many institutions engaged in livestock and aquaculture research [182,183].

## 5. Future Directions

**Figure 3:**
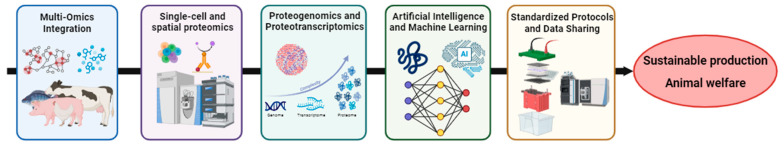
Emerging approaches in livestock proteomics for sustainable production and animal welfare. The figure highlights key methodologies, including multi-omics integration, single-cell and spatial proteomics, proteogenomics and proteotranscriptomics, artificial intelligence, and machine learning, as well as standardized protocols and data sharing, all contributing to improvements in sustainable production and animal welfare.

Proteomics is an increasingly valuable tool in aquaculture and livestock research, offering promising future directions that can advance our understanding of biological processes, improve production efficiency, and enhance health management. Integrating proteomics with other omics technologies and leveraging emerging innovations can enhance the benefit of groundbreaking discoveries and practical applications in the fields of aquaculture and livestock (Figure 3).

### 5.1. Multi-Omics Integration and Systems Biology Approaches

Integrating proteomics with genomics, transcriptomics, and metabolomics provides a comprehensive understanding of the physiological and molecular mechanisms underlying key traits such as growth, reproduction, and disease resistance in livestock and aquaculture. Multi-omics approaches in livestock have revealed how the milk microbiome modulates host physiology, improves genetic potential, and aids in disease prevention [184,185]. Similarly, combining proteomics with other omics tools in aquaculture enables researchers to track protein expression changes in fish exposed to environmental stressors such as high salinity or extreme temperatures, aiding in the identification of biomarkers for health monitoring and adaption [186,187]. Multi-omics studies in livestock have also assessed the effects of low-dose antibiotics (LDA) on hepatocellular functions, lipid metabolism, and immunity, revealing epigenetic and transcriptional changes critical to growth promotion [188]. Such approaches can be adopted in aquaculture to investigate the molecular effects of nutritional supplements, antibiotics, or environmental toxins, leading to targeted interventions to improve fish welfare and productivity.

### 5.2. Single-Cell and Spatial Proteomics

Single-cell and spatial proteomics are emerging as transformative technologies in aquaculture and livestock, enabling the investigation of cellular heterogeneity and spatially resolved protein expression patterns in unprecedented detail [177,189]. In livestock, single-cell proteomics has been applied to study immune responses, such as protein-level changes in neutrophils during uterine diseases in cattle, which can guide therapeutic developments [190]. Spatial proteomics, on the other hand, allows for the mapping of protein expression in tissues such as the liver or mammary glands, enhancing our understanding of metabolic functions and disease mechanisms [191]. In aquaculture, these approaches can be used to examine the localized immune responses in pathogen-infected fish tissues or to investigate protein changes in specific organs under environmental stress. Single-cell techniques could also aid in identifying novel therapeutic targets for fish diseases [10].

### 5.3. Proteogenomics and Proteotranscriptomics

Proteogenomics and proteotranscriptomics, which integrate proteomics with genomic and transcriptomic datasets, enable a more precise, functional annotation of genes and proteins [192,193]. These approaches have been employed in livestock to identify novel antimicrobial resistance genes and proteins associated with meat quality traits [194,195]. In aquaculture, proteogenomics can help decipher the complexity of fish biology by linking protein expression to phenotypic traits such as disease resistance and stress tolerance, enabling more effective breeding programs and health management strategies [196,197]. These insights can also aid in vaccine development, targeted drug delivery systems, and the creation of diagnostic tools for aquaculture species.

### 5.4. Artificial Intelligence in Proteomics

Artificial intelligence (AI) and machine learning (ML) are revolutionizing proteomics data analysis in both aquaculture and livestock. AI can process complex datasets, identify protein expression patterns, and build predictive models for disease progression or production traits [198,199]. In livestock, AI-based tools such as DIABLO have successfully integrated multi-omics datasets to identify biomarkers related to milk production efficiency and disease resistance [200]. Similarly, in aquaculture, AI could assist in predicting fish health outcomes, optimizing feed formulations, and identifying stress biomarkers for environmental monitoring [201]. These technologies also hold promise for real-time applications, including biosensors or microfluidic devices for rapid field diagnostics in both aquaculture and livestock production systems.

### 5.5. Standardized Protocols and Data Sharing

The advances in proteomics in aquaculture and livestock requires the development of standardized protocols for sample preparation, data acquisition, and analysis [202,203]. In livestock research, repositories such as mzTab, PRIDE, and PeptideAtlas have facilitated data sharing and cross-study comparisons, which are essential for validating findings and expanding the field [204]. However, a major challenge remains in the lack of universal protocols and species-specific reference databases for aquaculture. Developing such resources for fish species will facilitate cross-species comparisons and accelerate discoveries in fish biology and aquaculture management.

## 6. Conclusions

Advanced technologies such as proteomics, genomics, and real-time monitoring are revolutionizing precision livestock and aquaculture farming by enabling personalized management strategies, enhanced disease control, and sustainable practices. These innovations address consumer demand for ethical production and align with sustainability goals. However, challenges related to cost and scalability require further research and innovation. Ultimately, these advances have the potential to transform livestock and aquaculture into more sustainable, resilient, and ethically responsible industries.

## Data Availability

Data are contained within the article.
